# Neural Pattern Similarity in the Left IFG and Fusiform Is Associated with Novel Word Learning

**DOI:** 10.3389/fnhum.2017.00424

**Published:** 2017-08-22

**Authors:** Jing Qu, Liu Qian, Chuansheng Chen, Gui Xue, Huiling Li, Peng Xie, Leilei Mei

**Affiliations:** ^1^Guangdong Key Laboratory of Mental Health and Cognitive Science, Center for Studies of Psychological Application, School of Psychology, South China Normal University Guangzhou, China; ^2^Department of Psychology and Social Behavior, University of California, Irvine Irvine, CA, United States; ^3^State Key Laboratory of Cognitive Neuroscience and Learning, IDG McGovern Institute for Brain Research, Beijing Normal University Beijing, China

**Keywords:** pattern similarity, language training, reading, individual difference, fMRI

## Abstract

Previous studies have revealed that greater neural pattern similarity across repetitions is associated with better subsequent memory. In this study, we used an artificial language training paradigm and representational similarity analysis to examine whether neural pattern similarity across repetitions before training was associated with post-training behavioral performance. Twenty-four native Chinese speakers were trained to learn a logographic artificial language for 12 days and behavioral performance was recorded using the word naming and picture naming tasks. Participants were scanned while performing a passive viewing task before training, after 4-day training and after 12-day training. Results showed that pattern similarity in the left pars opercularis (PO) and fusiform gyrus (FG) before training was negatively associated with reaction time (RT) in both word naming and picture naming tasks after training. These results suggest that neural pattern similarity is an effective neurofunctional predictor of novel word learning in addition to word memory.

## Introduction

Cognitive neuroscientists are generally interested in identifying effective neural predictors of individuals’ learning ability. Up to now, a number of previous studies have found that preexisting individual differences in neural activity might serve as neurofunctional predictors of learning (Xue et al., 2006a; Chen et al., 2007; Wong et al., 2007; Asaridou et al., 2015; Chai et al., 2016; Kepinska et al., 2017). For example, in visual language learning, Xue et al. (2006a) revealed that interindividual variability in fusiform asymmetry before training was associated with visual word learning after 2-week training. In addition, Mei et al. (2008) found that preexisting individual differences in neural activity in the left posterior superior temporal sulcus predicted the efficiency in learning auditory words in a new language. These results suggest that preexisting individual differences in neural responses predict subsequent learning performance.

Nevertheless, previous studies mainly focused on the relation between activation intensity of brain regions and learning performance by using univariate analysis which may lose rich representational space information (Kriegeskorte and Kievit, 2013). Recently, using representational similarity analysis (Kriegeskorte et al., 2008) that computes multivoxel neural pattern, researchers started to investigate the relation between neural representational pattern and word memory and retention (Xue et al., 2010a, 2013; Davis et al., 2014; Lu et al., 2015; Wirebring et al., 2015; Xiao et al., 2016). Pattern similarity, indexed by the correlation in neural responses across repetitions of the same stimuli, is a widely-used measure for memory encoding (Xue et al., 2010a, 2013; Poh and Chee, 2017). Pattern similarity has been shown to be predictive of subsequent memory and is thought to reflect pattern reinstatement due to study-phase retrieval (Xue et al., 2010a, 2013; Lu et al., 2015). For instance, Xue et al. (2010a) found that, compared with forgotten items, subsequently remembered words showed greater pattern similarity across repetitions in frontoparietal and occipitotemporal regions, including (but not limited to) the regions whose mean activity were correlated with subsequent memory. Therefore, pattern similarity may be an effective marker of individuals’ memory encoding ability, and consequently predictive of the learning outcomes.

To examine the associations of neural pattern similarity and behavioral performance after novel word learning, the present study trained 24 Chinese college students to learn a logographic artificial language (created based on Korean Hangul) for 12 days. Participants were scanned while performing a widely used reading task (i.e., passive viewing) before training, after 4-day training and after 12-day training. The predictive role of neural pattern similarity was examined by correlating neural pattern similarity before training and reading performance after training. The left inferior frontal gyrus and fusiform gyrus (FG) were predefined as regions of interest (ROI) because of their crucial involvement in visual word processing and learning (Cohen and Dehaene, 2004; Dehaene and Cohen, 2011; Price and Devlin, 2011; Pinel et al., 2014). Specifically, in word reading, the left FG and inferior frontal gyrus are thought to be responsible for visual form processing and high-level language processing (e.g., phonological and semantic processing) during word reading, respectively (Poldrack et al., 1999; Gold et al., 2005; Dehaene and Cohen, 2011; Price, 2012; Wimmer et al., 2016). In word learning and memory, those two regions have also been consistently found to be involved in successful learning and memory of visual words (Mei et al., 2010; Xue et al., 2010a,b). Therefore, we hypothesized that neural pattern similarity before training was associated with learning outcomes after artificial language training.

## Materials and Methods

### Participants

Twenty-four native Chinese college students from South China Normal University (11 males and 13 females; mean age = 19.46 ± 0.93; range from 18 to 22) who have learned English as their second language and have no prior experience of Korean language were recruited for the study. Participants in the study had normal or corrected-to-normal vision, had no history of head injury or any diagnosis of psychiatric or neurological disorders, and were strongly right-handed as judged by Snyder and Harris’s handedness inventory (Snyder and Harris, 1993). The study was conducted according to the latest version of Declaration of Helsinki. Before the experiments, written informed consent was obtained for all participants. The present study was approved by the IRB of School of Psychology at South China Normal University.

### Materials

Thirty English words, thirty Chinese words and thirty artificial language words were used in the study. All English words were presented in gray-scale with 340 × 226 pixels in size, and Chinese words and the artificial language words were 226 × 151 pixels in size.

Chinese words were medium- to high-frequency single-character words according to a database of Chinese word and character frequencies (Cai and Brysbaert, 2010). On average, they occurred at the rate of 57 per million words. The words consist of 2–9 stokes. English words were selected from the MRC Psycholinguistic Database^1^. They were medium- to high-frequency words (mean = 54.63 per million, SD = 33.53), 3–6 letters (mean = 4.5, SD = 1.11) in length. The artificial language words were matched with Chinese words in visual complexity. The sounds of the artificial language words were recorded by a native Korean female speaker. All the sounds were denoised and normalized to the same length (600 ms) and loudness using Audacity 1.3^2^.

### Training Procedure

**Figure 1:**
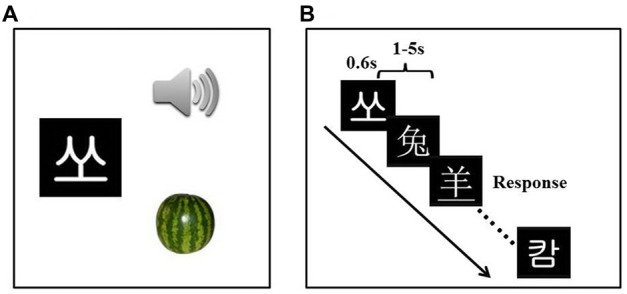
Experiment design and examples of the stimuli. Participants received the artificial language training **(A)** for 12 days (1 h per day). The passive viewing task **(B)** was administered three times.

We trained participants in 12 learning sessions with one session (roughly 1 h) per day. Participants were asked to learn the associations of visual forms, sounds and semantics of 30 artificial language words using a computerized program. The artificial language was created by adopting the visual forms and sounds of 30 Korean Hangul characters, which were assigned arbitrary meanings through pictures of 30 different objects (see Figure 1A).

We used a combination of tasks to facilitate efficient learning. The tasks included character learning (associating each artificial language visual word with its sound and meaning), phonological choice task (choosing the correct sound out of four to match the target word), semantic choice task (choosing the correct meaning out of four to match the target word), free learning (re-learning any words with which participants had difficulties in the phonological/semantic choice tasks), naming with feedback (reading a word aloud followed by a feedback with its correct pronunciation), fast matching (matching 10 visual words with 10 pictures as fast and accurately as possible) and fast naming (reading 10 words or naming 10 pictures as fast and accurately as possible).

### Behavioral Tasks

At the end of each training day, word naming and picture naming tasks were used to test the learning results. In both tasks, each artificial language word (in the word naming task) or each picture (in the picture naming task) was presented for 4 s (Days 1–4) or 3 s (after Day 4), followed by a 1 s blank. Participants were asked to read the artificial language word or to name the object on the picture aloud in the artificial language as fast and accurately as possible. The oral responses in those two tasks were recorded and each response’s accuracy was evaluated by a research assistant by comparing the participants’ responses with the pronunciations used for training.

To quantify the rate of learning, a learning curve was fitted to each participant’s naming speed data for the 12 learning sessions. To improve the stability of behavioral data, naming speed for each day was the average of the reaction times (RT) of the two naming tasks (i.e., word naming and picture naming tasks). A power function (*y* = a*x^−b^) was then used to fit the non-linear learning curve of RT for each participant, where *a* represents initial performance and *b* represents the rate of learning (Anderson, 1983; Logan et al., 1988). Larger *b* indicates faster learning. The goodness-of-fit was determined by the coefficient of determination.

### fMRI Task

Participants were scanned three times, one before training, one after 4-day training and one after 12-day training. A passive viewing task was performed in all three scans. Rapid event-related design was used for the passive viewing task. It consisted of three types of stimuli: English, Chinese and artificial language words. English materials were included to address other research questions, and thus excluded from data analysis in this article. Each type of materials contained 30 items and each item was presented twice. The two presentations of the same item were spaced by 4–8 other items (mean = 5.99). Stimulus presentation and response collection were programmed using Matlab (Mathworks) and the Psychtoolbox^3^ on a computer. Trial sequences were optimized with OPTSEQ^4^ (Dale, 1999).

During the scan, each stimulus was presented for 600 ms, with a jittered inter-stimulus interval varying randomly from 1 s to 5 s (mean = 2 s) to improve the design efficiency (Figure 1B; Dale, 1999). Participants were asked to carefully view the stimuli. To ensure that participants were awake and attentive, they were instructed to press a key whenever they noticed that the visual word was underlined (fillers). Participants correctly responded to 8.63 ± 0.65 of nine underlined words at the pre-training stage, 8.17 ± 1.61 at the mid-training stage (after Day 4) and 8.67 ± 1.43 at the post-training stage (after Day 12), suggesting participants were attentive to the stimuli during the passive viewing task. In total, the passive viewing task consisted of 189 trials (180 words and 9 fillers) and lasted for 9 min 26 s (283 TRs).

### MRI Data Acquisition

MRI image data were acquired with a 3.0 T Siemens MRI scanner in the MRI Center at South China Normal University. A single-shot T2*-weighted gradient-echo EPI sequence was used for functional imaging acquisition with the following parameters: TR/TE/θ = 2000 ms/25 ms/90°, FOV = 192 × 192 mm, matrix = 64 × 64 and slice thickness = 3.5 mm. Thirty-five contiguous axial slices parallel to the AC-PC line were obtained to cover the whole cerebrum and partial cerebellum. Anatomical MRI was acquired using a T1-weighted, three-dimensional, gradient-echo pulse-sequence. Parameters for this sequence were: TR/TE/θ = 2300 ms/3.24 ms/9°, FOV = 256*256 mm, matrix = 256*256, and slice thickness = 1 mm. One-hundred and seventy-six sagittal slices were acquired to provide a high-resolution structural image of the whole brain.

### Image Preprocessing and Statistical Analysis

Initial analysis was carried out using tools from the FMRIB’s software library^5^ version 4.1.2. The first three volumes in each time series were automatically discarded by the scanner to allow for T1 equilibrium effects. The remaining images were then realigned to compensate for small head movements (Jenkinson and Smith, 2001). Translational movement parameters never exceeded 1 voxel in any direction for any participant or learning session. All data were spatially smoothed using a 5-mm full-width-half-maximum Gaussian kernel. The smoothed data were then filtered in the temporal domain using a nonlinear high-pass filter with a 60-s cutoff. A 2-step registration procedure was used whereby EPI images were first registered to the MPRAGE structural image, and then into standard (Montreal Neurological Institute [MNI]) space, using affine transformations with FLIRT (Jenkinson and Smith, 2001) to the avg152 T1 MNI template.

At the first level, the data were fitted with a general linear model within the FILM module of FSL for each participant and each learning session. Events were modeled at the time of the stimulus presentation. The events’ onsets and durations were convolved with the canonical hemodynamic response function (double-gamma) to generate the regressors used in the general linear model. Temporal derivatives and the six motion parameters were included as covariates of no interest to improve statistical sensitivity. Null events (i.e., fixation) were not explicitly modeled, and therefore constituted an implicit baseline. The underlined words and English words were modeled as nuisance variables to avoid potential confounding effects. In total, four conditions of interest (i.e., the two repetitions of Chinese words and two repetitions of artificial language words) were modeled. The contrast image of each condition and of each repetition was computed separately for each learning session and for each participant.

A second-level analysis was performed on the three scans to compute the training effects for each participant, using a fixed-effects model. The training effects were computed by using three contrasts: [artificial language words—Chinese words] after 4 days of training—[artificial language words—Chinese words] before training, [artificial language words—Chinese words] after 12 days of training—[artificial language words—Chinese words] before training, and [artificial language words—Chinese words] after 12 days of training—[artificial language words—Chinese words] after 4 days of training. Then, the data from the second-level analyses were averaged across the participants in the third-level analysis, using a random-effects model (treating participants as a random effect) with FLAME stage 1 only (Beckmann et al., 2003; Woolrich et al., 2004; Woolrich, 2008). Unless otherwise indicated, group images were thresholded with a height threshold of *z* > 2.3 and a cluster probability, *p* < 0.05, corrected for whole-brain multiple comparisons using the Gaussian random field (GRF) theory (Worsley, 2001).

### Representational Similarity Analysis and Region of Interest Analysis

In this analysis, we first re-estimated the above mentioned first-level models with unsmoothed data. As mentioned above, four conditions (i.e., the two repetitions of Chinese words and two repetitions of artificial language words) were modeled. Then we extracted the contrast of parameter estimates (COPE) value from each voxel within the pre-defined ROIs using the fslmeants command, separately for each condition (Xue et al., 2010a). In other words, we extracted averaged voxel-wise activation across all stimuli for each condition. As noted in “Introduction” Section, four regions (the left pars opercularis (PO) and pars triangularis (PT) and two regions in the occipitotemporal areas; the bilateral FG) were defined as ROIs because of their critical role in visual word processing and encoding (Cohen and Dehaene, 2004; Mei et al., 2010; Xue et al., 2010a,b; Dehaene and Cohen, 2011; Weber et al., 2016). The ROIs were anatomically defined based on Harvard-Oxford probabilistic atlas (Maximal Probability Threshold: 25%) within FSL. Following previous studies (Xue et al., 2010a; Wirebring et al., 2015; Poh and Chee, 2017), we used Pearson correlation to compute pattern similarity across the two repetitions on the averaged activation patterns separately for Chinese and artificial language words. These correlation coefficients were transformed into Fisher’s *z*-scores. Correlational analysis was conducted to examine the association between pattern similarity before training and behavioral performance after training.

The associations between pattern similarity and behavioral performance were further tested by using between-subject and within-subject permutation tests, separately for each ROI and for each naming task (i.e., word naming and picture naming tasks). In the between-subject permutation test, pattern similarity of artificial language words in each ROI was shuffled across the 24 participants. In the within-subject permutation test, we shuffled pattern similarity for each ROI between the two conditions (i.e., Chinese words and artificial language words) for each participant. The permutated data were then correlated with the subjects’ behavioral performance on the two naming tasks (i.e., word naming and picture naming tasks). The permutation test was conducted 5000 times to obtain the distribution of correlation coefficients for each ROI and each task. The maximum number of possible combinations of two variables for the between-subject permutation of 24 participants was 24 factorial (24!), whereas the maximum number of possible combinations for the within-subject permutation test was 2^24^.

Second, to confirm the robustness of our results, we examined the pattern similarity of artificial language words after dividing them into high- and low-RT groups. Specifically, we divided the 30 artificial language words into two groups via median split on averaged RT from Day 5 to Day 12. The grouping was done twice, once using the RT from the word naming task, and the other time using RTs from the picture naming task. The two repetitions for the two groups of artificial language words were separately modeled. Unsmoothed data were used. All other parameters were the same as the above-mentioned first-level models. We then separately computed the pattern similarity for artificial language words with faster naming speed and for those with slower naming speed in the four ROIs using the same procedure as above. All similarity scores were Fisher-transformed. The differences between the groups of artificial language words were examined by using paired *T* tests.

Finally, we extracted the activation level (percent signal change) for the four ROIs. The percent signal changes in the four ROIs were calculated by extracting parameter estimates (betas) of each event type from the fitted model and averaging them across all voxels in the cluster for each participant. Percent signal changes were calculated using the following formula: [contrast image/(mean of run)] × ppheight × 100%, where ppheight was the peak height of the hemodynamic response vs. the baseline level of activity (Mumford, 2007).

## Results

### Training Improved the Behavioral Performance during Training

**Figure 2:**
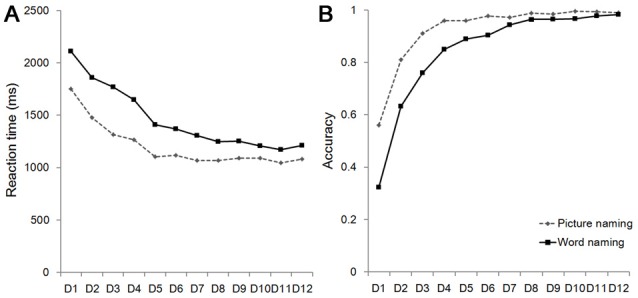
Reaction time (RT; **A**) and accuracy **(B)** on the word naming and picture naming tasks. D, Day.

As shown in Figure 2, RT for the picture naming and the word naming tasks significantly decreased as a result of training (the picture naming task: *F*_(11,253)_ = 65.377, *p* < 0.001; the word naming task: *F*_(11,253)_ = 78.274, *p* < 0.001). Training also resulted in increased accuracy in both the picture naming task (*F*_(11,253)_ = 60.084, *p* < 0.001) and the word naming task (*F*_(11,253)_ = 78.274, *p* < 0.001). These results suggest that our training was effective.

### Training Enhanced Activations in the Left Inferior Frontal Gyrus

**Figure 3:**
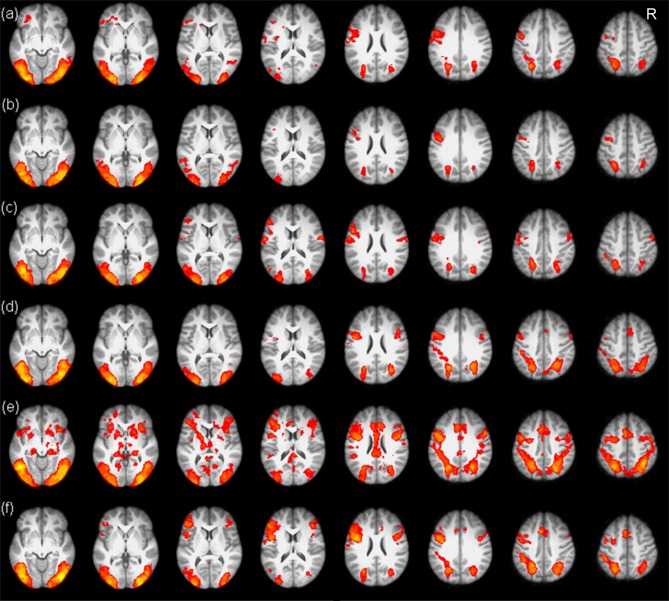
Brain maps of Chinese and artificial language word processing in the three scans. The upper three panels show brain activations for Chinese word processing before training **(A)**, after 4-day training **(B)** and after 12-day training **(C)**. The lower three panels show brain activations for artificial language word processing before training **(D)**, after 4-day training **(E)** and after 12-day training **(F)**. R, right.

The whole brain analysis revealed that the typical reading network, including the bilateral prefrontal cortex, the occipitoparietal cortex and the occipitotemporal cortex, was involved in the processing of both Chinese and artificial language words (see Figure 3).

**Figure 4:**
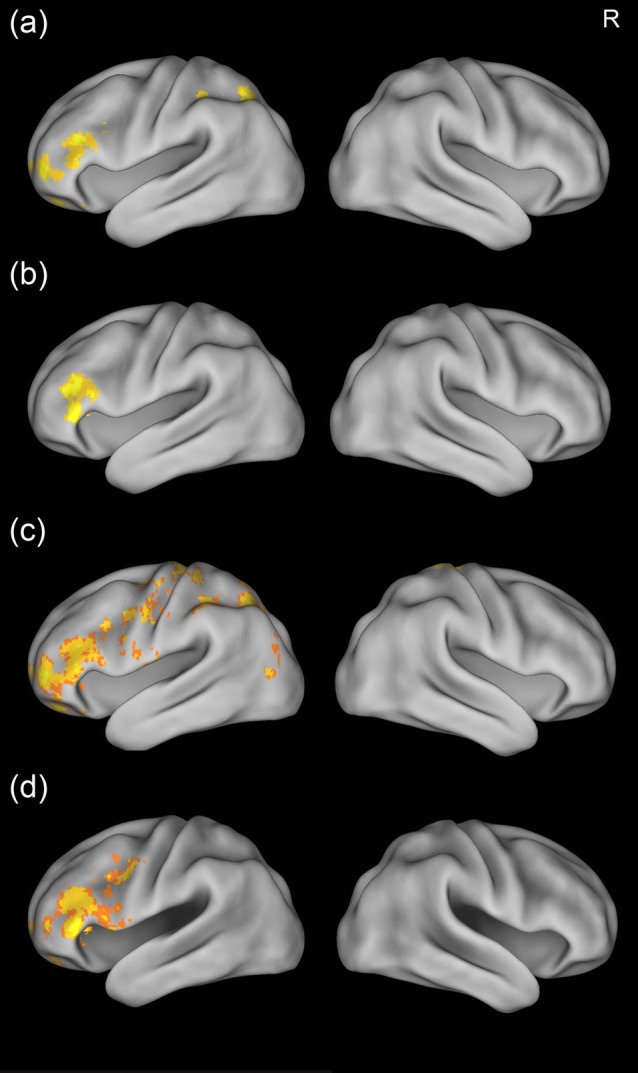
Training effects for the artificial language words. The upper two panels show brain maps corrected using the Gaussian random field (GRF) theory, while the lower two panels show brain maps corrected using non-parametric permutation test, There was greater activation in the left inferior frontal gyrus and superior parietal cortex for the mid-training scan than for the pre-training scan **(A,C)** and greater activation in the left inferior frontal cortex for the post-training scan than for the pre-training scan **(B,D)**. R, right.

We then examined the training effects by comparing the brain activation across the three scans. Results showed that there was stronger activation in the left inferior frontal gyrus for the mid-training (Day 4) scan and post-training (Day 12) scan than for the pre-training scan (Figures 4A,B). In addition, greater activation in the superior parietal cortex was found for the mid-training scan than for the pre-training scan. No region showed more activation for the pre-training scan than for the mid-training or the post-training scan. Because the GRF-based cluster statistics used in this study might have inflated false positive rates (Eklunda et al., 2016), we performed an additional analysis by using a randomization procedure (FSL’s randomize, non-parametric permutation test) repeated 5000 times to examine the training effects (corrected, *p* < 0.05). The results of GRF-based cluster statistics were confirmed by the non-parametric permutation test (Figures 4C,D).

### Pattern Similarity before Training Was Positively Associated with Naming Speed

We further conducted several analyses to examine whether pattern similarity of artificial language words across the two repetitions before training was associated with behavioral performance after training. For both the word naming and picture naming tasks, the behavioral performance was calculated by averaging the last 8 days’ RT (i.e., Day 5–12), because the learning curves became smooth after Day 5 for both RT and accuracy in both tasks (Figure 2). Accuracy was excluded in this analysis because of its smaller variances (i.e., the ceiling effect).

**Figure 5:**
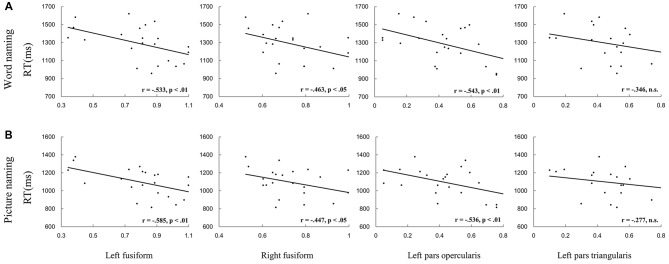
Pattern similarity before training predicted RT in the word naming **(A)** and picture naming **(B)** tasks. Results showed that pattern similarity in the left pars opercularis (PO) and bilateral fusiform gyrus (FG) predicted RT in both naming tasks.

First, we correlated the pattern similarity in the four ROIs (i.e., the left PO, PT and bilateral FG) before training with behavioral performance, respectively. As expected, for both word naming and picture naming tasks, we found that pattern similarity of artificial language words before training was associated with RT after training. Significant negative correlations were found in the left PO and bilateral FG (see Figure 5 and Table 1). No regions showed significant correlation between pattern similarity of Chinese words and artificial language’s naming speed (Table 1). The differences in correlation coefficients of the two types of words were further examined by using Fisher’s *r*-to-*z* transformation test with one variable in common (Lee and Preacher, 2013). Results showed that the correlation coefficients were significantly greater for artificial language words than for Chinese words in the left PO (*z* = −1.83, *p* < 0.05, one-tailed) and bilateral FG (left: *z* = −2.93, *p* < 0.01; right: *z* = −2.46, *p* < 0.01, one-tailed) in the word naming task and in the bilateral FG in the picture naming task (left: *z* = −2.98, *p* = 0.001; right: *z* = −1.70, *p* < 0.05, one-tailed). In addition, we examined the correlations between pattern similarity after training and behavioral performance. Similar to the results before training, pattern similarity in the left fusiform cortex after training was negatively correlated with behavioral performance, although the correlations of pattern similarity after 12-day training were not statistically significant (Table 2).

**Table 1:** Correlations between pattern similarity before training and reaction time (RT) in the two naming tasks (*p < 0.05 and **p < 0.01).

ROI	Word naming		Picture naming	
	*r*	*p*	*r*	*p*
**Artificial language word**				
Left fusiform	−0.533	0.007**	−0.585	0.003**
Right fusiform	−0.463	0.023*	−0.447	0.029*
Left pars opercularis	−0.543	0.006**	−0.536	0.007**
Left pars triangularis	−0.346	0.097	−0.277	0.190
**Chinese word**				
Left fusiform	−0.106	0.623	−0.165	0.442
Right fusiform	−0.010	0.962	−0.133	0.536
Left pars opercularis	−0.224	0.293	−0.308	0.143
Left pars triangularis	−0.026	0.903	0.076	0.725

**Table 2:** Correlations between pattern similarity at Day 4 and Day 12 and RT in the two naming tasks (*p < 0.05).

ROI	Word naming		Picture naming	
	*r*	*p*	*r*	*p*
**Day 4**				
Left fusiform	−0.488	0.016*	−0.434	0.034*
Right fusiform	−0.270	0.201	−0.362	0.082
Left pars opercularis	−0.281	0.183	−0.338	0.106
Left pars triangularis	−0.344	0.100	−0.450	0.027*
**Day 12**				
Left fusiform	−0.375	0.071	−0.388	0.061
Right fusiform	−0.381	0.066	−0.460	0.024*
Left pars opercularis	−0.186	0.383	−0.283	0.180
Left pars triangularis	−0.244	0.251	0.461	0.023*

**Figure 6:**
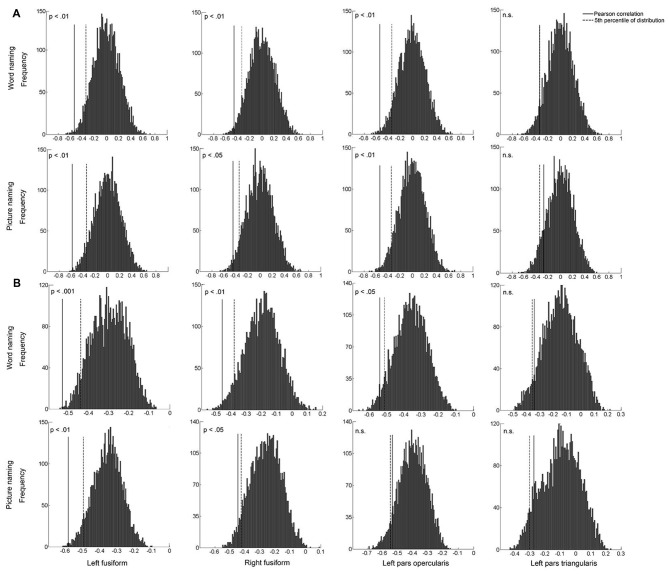
The histogram plots of between-subject permutation test **(A)** and within-subject permutation test **(B)**. The solid line indicates the actual correlation between pattern similarity of artificial language words and behavioral performance, and the dashed line indicates the 5th percentile (0.05) of the distribution. *X*-axis represents the correlation coefficients.

To confirm the robustness of the above associations, we conducted between-subject and within-subject permutation tests, separately for each ROI and for each naming task. The between-subject permutation test revealed that the associations in the left PO and bilateral FG were significant in both naming tasks (all *p*s < 0.05; Figure 6A). Similar results were found for the within-subject permutation test. Specifically, the correlations in the bilateral FG were significant in both naming tasks. The correlation in the left PO was additionally significant in the word naming task (Figure 6B).

**Figure 7:**
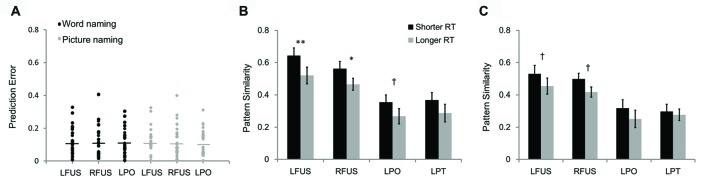
The scatter plot **(A)** shows prediction errors for leave-one-out cross-validation. The two bar graphs show differences in pattern similarity between artificial language words with longer RT and those with shorter RT in word naming **(B)** and picture naming tasks **(C)**. LFUS, left fusiform; RFUS, right fusiform; LPO, left pars opercularis; and LPT, left pars triangularis. **p < 0.01, *p < 0.05, ^†^*p* < 0.10.

We also tested the predictive power of pattern similarity before training on behavioral performance by using leave-one-out cross-validation. In this analysis, the brain-behavior regression model was estimated based on 23 subjects and tested on the remaining one subject. The prediction error was calculated by using the formula of (predicted RT − observed RT)/observed RT, which represents the deviation percentage of the predicted RT from observed RT. Results showed that the prediction errors were less than 0.33 for the pattern similarity in the left PO (word naming: 0.004–0.305; picture naming: 0.002–0.311) and fusiform cortex (word naming: 0.007–0.328; picture naming: 0.004–0.326; Figure 7A). The predictive power was a little lower in the right fusiform cortex, whose maximum prediction errors reached 0.406 (word naming: 0.016–0.406; picture naming: 0.007–0.400).

The results of between-subject brain-behavior correlations were further confirmed by within-subject analysis by comparing pattern similarity of artificial language words with high- and low-RTs. Consistent with the brain-behavior correlational analysis reported above, pattern similarity in the bilateral fusiform cortex significantly differed between the two groups of words divided based on RT for the word naming task (Figure 7B). Specifically, pattern similarity was greater for artificial language words with faster naming speed than those with slower naming speed (left fusiform: *t*_(23)_ = 3.05, *p* < 0.01; right fusiform: *t*_(23)_ = 2.27, *p* < 0.05). There was a similar trend for pattern similarity in the left PO (*t*_(23)_ = 1.89, *p* = 0.071). Similar results were found for the two groups of words divided based on RT in the picture naming task (Figure 7C). Specifically, words with shorter RT in the picture naming task showed marginally greater pattern similarity in the bilateral fusiform cortex than words with longer RT (left fusiform: *t*_(23)_ = 1.96, *p* = 0.062; right fusiform: *t*_(23)_ = 1.99, *p* = 0.059).

As noted in “Introduction” Section, previous studies have found that activation in those regions also predicts language learning (Xue et al., 2006a; Asaridou et al., 2015). Therefore, we also performed correlational analysis to examine whether the activation level (percent signal change) in the four ROIs before training was associated with behavioral performance after training. Consistent with previous study (Asaridou et al., 2015), we found that the activation level in the PO was negatively associated with RT in both naming tasks (Table 3).

**Table 3:** Correlations between activation level before training and RT in the two naming tasks (*p < 0.05 and **p < 0.01).

ROI	Word naming		Picture naming	
	*r*	*p*	*r*	*p*
Left fusiform	−0.265	0.211	−0.315	0.134
Right fusiform	−0.280	0.185	−0.399	0.054
Left pars opercularis	−0.458	0.024*	−0.529	0.008**
Left pars triangularis	−0.162	0.450	−0.123	0.567

**Figure 8:**
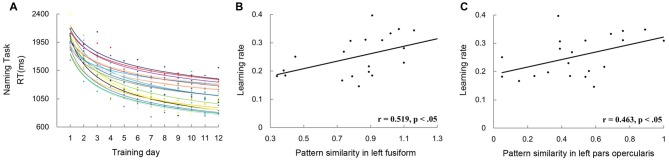
The left graph **(A)** shows the scatter plot and fitted learning curve for each participant. The naming speed in each training day was calculated by averaging the RTs of the word and picture naming tasks. The two scatter plots on the right show correlations between learning rate and pattern similarity of artificial languages words in the left FG **(B)** and PO **(C)**.

Finally, to rule out the possibility that the correlations found in this study reflected the association between pattern similarity and general naming speed, but not novel word learning, we conducted correlational analysis on pattern similarity and the rate of learning. Data of three participants were excluded in this analysis because of their relatively poor goodness-of-fit (*R*^2^ < 0.7). The goodness-of-fit was 0.87 ± 0.07 for the remaining participants (Figure 8A). Consistent with the results of naming speed, significant correlations between pattern similarity and the rate of learning were found in the left PO (*r* = 0.519, *p* < 0.05; Figure 8B) and FG (*r* = 0.463, *p* < 0.05; Figure 8C).

## Discussion

Using an artificial language training paradigm and representational similarity analysis, we examined the associations between neural pattern similarity before novel word learning and behavioral performance after training. Consistent with previous language learning studies (Xue et al., 2006b; Mei et al., 2014), we found that training increased activation in the prefrontal cortex and parietal cortex because of increased demands of semantic and phonological processing after training. More importantly, using the pattern similarity analysis, we found that pattern similarity across repetitions before training was correlated with the learning performance after 12 days of training. Specifically, greater pattern similarity in the left PO and FG was associated with better learning outcomes (i.e., faster naming speed). These results were further confirmed by the direct comparisons of artificial language words with high and low naming speeds. These results suggest that neural pattern similarity is an effective neurofunctional predictor of novel word learning.

Previous studies have identified neurofunctional predictors by correlating activation intensity before training with learning outcomes after training (Xue et al., 2006a; Chen et al., 2007; Mei et al., 2008; Asaridou et al., 2015). However, the univariate activation analysis typically uses neural activity in one voxel or overall activation in one region, and consequently misses the information of multi-voxel neural pattern. Using representational similarity analysis (Kriegeskorte et al., 2008; Xue et al., 2010a) that computes multi-voxel pattern similarity, we found that pattern similarity in the left inferior frontal gyrus and bilateral FG before training predicted learning outcomes after training. Specifically, greater pattern similarity was associated with better learning outcomes. Furthermore, these associations were only significant for pattern similarity of artificial language words, but not for that of words in other languages. These results extend the previous finding of neural pattern similarity’s association with subsequent memory (Xue et al., 2010a, 2013; Davis et al., 2014; Xiao et al., 2016) to the new finding of its association with long-term novel word learning.

The associations between neural pattern similarity and behavioral performance found in this study may involve at least two possible mechanisms. First, pattern similarity has been shown to be predictive of subsequent memory and is thought to reflect pattern reinstatement due to study-phase retrieval (Xue et al., 2010a, 2013). It benefits memory encoding through the provision of consistent input (Xue et al., 2013; Lu et al., 2015; Poh and Chee, 2017). Although the passive viewing task used in this study did not require participants to explicitly memorize the words, there is evidence that pattern reinstatement occurs even without explicit requirement of memory (Xue et al., 2010a, 2013). From this perspective, the level of pattern similarity in this study might represent individuals’ ability of memorizing artificial language words, and consequently was associated with behavioral performance after subsequent artificial language word learning. Nevertheless, unlike previous studies (Xue et al., 2010a, 2013), pattern similarity was computed on averaged activation patterns in this study because of its rapid event-related design. Averaged activation patterns might lose unique pattern information. Therefore, future studies should test the pattern reinstatement account by adopting a slow event-related design to separate the pattern similarity of individual memories. Second, pattern similarity may be a more general marker of effective cognitive processing. It has been shown that more reproducible neural patterns are associated with more conscious cognitive processing (Schurger et al., 2010). Our results seems to disconfirm the second explanation because pattern similarity of Chinese words was not correlated with behavioral performance of artificial language learning.

Our results of the predictive role of the left inferior frontal gyrus and fusiform cortex in novel word learning are consistent with previous findings of their crucial involvement in language processing and learning (Dehaene et al., 2002, 2010; Dehaene and Cohen, 2011; Asaridou et al., 2015). Particularly, the fusiform cortex is repeatedly reported in visual word processing. It has been proposed to be responsible for visual form processing (Dehaene et al., 2002; Cohen and Dehaene, 2004) or integrating low-level visuospatial features with higher-level associations (Price and Devlin, 2011). The left inferior frontal gyrus also plays an important role in language processing. It is thought to be involved in a variety of language processes, such as phonological (Poldrack et al., 1999; Taylor et al., 2013), semantic (Poldrack et al., 1999; Price, 2012), and syntactic processing (Henderson et al., 2016; Kuhnke et al., 2017; Matchin et al., 2017). Furthermore, the two regions have been found to be involved in successful memory of visual words (Wagner et al., 1998; Mei et al., 2010; Xue et al., 2010a) and predictive of visual word learning (Xue et al., 2006a; Chen et al., 2007; Dong et al., 2008). Our results confirmed the crucial involvement of those two regions in visual word processing and learning.

Two limitations should be discussed. First, the artificial language used in this study is different from natural language in several aspects, such as a limited vocabulary size and a lack of morphology and syntax. Such differences might limit the generalization of our findings to natural languages to some extent, although researchers have found high positive correlations between natural language learning and artificial language learning (Ettlinger et al., 2016). Therefore, future studies should confirm the predictive role of pattern similarity in natural language learning. Second, pattern similarity was computed across the two repetitions on the averaged activation patterns for all words, because we used a rapid event-related design which did not allow us to precisely estimate the neural responses of single trials. Therefore, our study was not able to separate the contributions of within-item pattern similarity and between-item pattern similarity in novel word learning. Future studies should use a slow event-related design like previous studies (Xue et al., 2010a; Poh and Chee, 2017) to examine the associations between the two types of pattern similarity and learning outcomes.

In sum, using the artificial language training paradigm and representational similarity analysis, this study revealed that greater pattern similarity across repetitions before training was associated better learning outcomes. These results suggest that pattern similarity is an effective neurofunctional predictor for novel word learning.

## Author Contributions

JQ, CC, GX and LM designed research. JQ, LQ, HL and PX performed research. JQ, LQ and LM analyzed the data. JQ, LQ, CC, GX, HL, PX and LM wrote and approved the article.

## Conflict of Interest Statement

The authors declare that the research was conducted in the absence of any commercial or financial relationships that could be construed as a potential conflict of interest.

## Footnotes

^1^http://websites.psychology.uwa.edu.au/school/MRCDatabase/uwa_mrc.htm

^2^http://www.audacity.sourceforge.net/

^3^www.psychtoolbox.org

^4^http://surfer.nmr.mgh.harvard.edu/optseq/

^5^www.fmrib.ox.ac.uk/fsl
